# Birds Drinking Alcohol: Species and Relationship with People. A Review of Information from Scientific Literature and Social Media

**DOI:** 10.3390/ani10020270

**Published:** 2020-02-09

**Authors:** Piotr Tryjanowski, Mateusz Hetman, Paweł Czechowski, Grzegorz Grzywaczewski, Petr Sklenicka, Klaudia Ziemblińska, Tim H. Sparks

**Affiliations:** 1 Institute of Zoology, Poznań University of Life Sciences, Wojska Polskiego 71 C, 60-625 Poznań, Poland; gerwazyjas@gmail.com (M.H.); thsparks@btopenworld.com (T.H.S.); 2 Faculty of Environmental Sciences, Czech University of Life Sciences Prague, Kamýcká 129, 165 00 Prague 6, Czech Republic; sklenicka@fzp.czu.cz; 3 Branch Faculty of the University of Zielona Góra in Sulechów, Armii Krajowej Str. 51, 66-100 Sulechów, Poland; 4 Department of Zoology and Animal Ecology University of Life Sciences in Lublin, Akademicka 13,20-950, Lublin, Poland; grzegorz.grzywaczewski@up.lublin.pl; 5 Department of Meteorology, Poznan University of Life Sciences; Piątkowska 94, 60-649 Poznań, Poland; klaudiaziem@wp.pl; 6 Museum of Zoology, University of Cambridge, Downing Street, Cambridge CB2 3EJ, UK

**Keywords:** anthropogenic impact, alcohol abuse, junk food, social media, YouTube

## Abstract

**Simple Summary:**

Alcohol consumption is quite common in the bird world. Using scientific literature and Internet resources (available because of the rising popularity of social media), we investigate which species and sources of ethanol are most frequently used by captive and wild birds. Moreover, it was possible with the internet videos to discriminate between intentional and unintentional ingestion. This information may be helpful in choosing appropriate species for future laboratory studies about ethanol metabolism in birds and their behaviour.

**Abstract:**

Ethanol is a natural by-product of the fermentation process of fruit sugars and is occasionally consumed by fruit-eating and tree sap drinking birds. Information on this form of alcohol consumption features in the scientific literature. However, as pets or as wild animals living close to humans in urban habitats, birds have increasing possibilities to consume alcohol from beverages, such as beer, wine or spirits. Some observations have been discussed in a light-hearted manner in mass media and social media, but without any generalization of why some bird species drink the beverages intentionally or unintentionally provided by humans. To check which species and in what circumstances birds drink alcohol and how this is evaluated by humans, we reviewed the scientific literature and analysed videos from YouTube. In total we found and analysed 8 scientific papers and 179 YouTube videos, from which we identified at least 55 species (in some cases not all birds were identified to species level), 11 in the scientific literature and 47 in videos. The distribution of these species over the avian phylogenetic tree suggests that the origin of this convergent behaviour is mainly by human influence. The two data sources differed in the species covered. Videos typically presented interactions of birds with human-provided alcoholic beverages, and were dominated by two groups of intelligent birds: parrots and corvids. The popularity of YouTube videos for a particular species was positively correlated with the general popularity of the species as measured by the number of hits (results listed) on Google. Human responses to the videos were generally very positive and we analysed how the responses were influenced by factors derived from viewing the videos. Moreover, YouTube videos also provide information on at least 47 new bird species not previously mentioned as using alcohol, and our results suggest that parrots in particular can be potentially good candidates for future restricted laboratory studies on the effect of ethanol on birds and their relationship with humans.

## 1. Introduction

People, but also some other animals, have been attracted by ethanol, a natural by-product of the fermentation process of fruit sugars, for millennia [1,2,3]. Currently, most research focuses on the problem of alcohol abuse, although other issues that have an evolutionary background are also scientifically interesting [1,4]. For example, ethanol has been shown to stimulate appetite and increase energy intake in humans and the same could be the case for limited alcohol consumption in birds [5]. In addition, ethanol represents a direct nutritional reward because its calorific value is nearly double that of carbohydrates [5,6]. Therefore, birds use alcohol from ripe fruit, especially berries [7], and tree sap [8] and ethanol consumption may both negatively and positively affect birds, which may perhaps be especially important in harsh winter conditions [9]. Experimental testing in European starling *Sturnus vulgaris*, has shown that ethanol can be degraded in the blood of birds [10], and affect singing behaviour, and then mating behaviour, for example in zebra finch *Taeniopygia guttata* [11]. 

However, alcohol provided intentionally and unintentionally by humans in alcoholic beverages, such as beer, wine and spirits, may also currently be very available to birds. For example, very often ethanol is consumed together with food, sometimes junk food, that affects the gut microbiota of birds [12]. Birds are readily attracted by human food and drink, and cases of alcohol abuse by birds are especially widely reported and commented upon in the mass media (Supplementary Materials) [13,14]. However, they often describe this phenomenon in a tabloid sensational style and unfortunately very often with a questionable identification of the bird species. However, this raises the interest of the general public, although because of the individual nature of the cases and the format of transmission, it has not hitherto encouraged scientific inquiry. On the other hand, the massive use of smartphones and uploading of videos on the YouTube platform offer possibilities to check occurrences of alcohol consumption by birds and show it in a social context. These videos can provide additional sources of information to those from a classical review of published papers, because they present available raw data in the form of uploaded videos, and thus offer options for internal validity, large samples and free and ubiquitous availability [15,16]. Video sharing platforms allow insights into how people perceive their pets inside their homes, and likewise the interactions that are commonly experienced with animals in the wild [16].

Therefore, the objectives of the current study were to check (1) which bird species consumed alcohol; (2) how the scientific literature and social media differ in mentioned species and methods; (3) under what circumstances—from analyses of YouTube videos—(place, kind of drink, flocking) birds consume alcoholic beverages; (4) the potential link between the overall popularity of a particular bird species [17,18] and the number of reported cases of birds consuming alcoholic drinks; (5) how humans feel about watching videos of drinking birds. Finally, we discuss how taking alcoholic beverages from humans can be similar to the innovative behaviour of some birds [19] and why future laboratory studies have to include more species in analyses.

## 2. Materials and Methods 

We carried out a comprehensive bibliographic search of publications, using the Google Scholar database to search for scientific articles sometimes not reported in more restricted databases, such as Scopus or Web of Science. For searching we used many combinations of keywords including bird*, avian and alcohol* (and different kind of drinks, e.g., beer, wine, vodka etc.) during June 2019, but with no filter for publication date. Subsequently, in each article that we found we searched all the references for other published papers which might also contain data useful for our review. From each scientific paper we noted information on the form of alcohol used by birds, a description of methods and the study species. Although some information can come from electronic mass media, such as blogs, after checking many of them we decided to exclude these data, because very often they cover non-creditable information on species and situations (SI), for example illustrated by freely available photos from other sources or only mentioning information obtained from members of the public without any detail. Moreover, they were often simply popular stories based on already published scientific papers.

However, the most important part of our study was conducted on materials that we collected by exploring internet resources, including recorded videos, that contained information for further analyses. Firstly, in June 2019, we used the YouTube and Dailymotion video platforms, then Facebook, to find videos of birds consuming or encouraged to ingest alcohol. Only good quality video recordings including details that allowed the identification of the bird species were used further. Videos were uploaded by bird owners, news channels, random bird observers or channels specialising in humorous content which were detected by specific terms in the name such as “viral” “funny” etc.

To search for data, we used key words such as “bird”, “parrot”, and the colloquial name of species, e.g., “budgie”, accompanying the words “alcohol”, “drunk”, “beer”, “vodka”, “wine”, in English and also, using Google Translate, other languages such as Spanish, Portuguese and Russian. 

Altogether, we collected 179 (176 on YouTube, 2 Facebook, 1 Dailymotion) video recordings which featured birds ingesting alcohol. Furthermore, we collected, wherever possible, data characterising each individual video including species identification and seven other variables: (1) type of alcohol, (2) interaction with people, (3) environment, (4) number of birds, (5) type of bird, (6) type of video channel, (7) species of bird and (8) social responses to the video. Firstly, we discriminated the type of consumed alcohol (1) between beer, wine, distilled alcoholic beverages and highly fermented fruits. Because of small samples in the latter categories this was recategorized for analysis as beer and other. The next step was evaluation of bird–human interactions (2), i.e., was alcohol unintentionally or intentionally provided by humans. We distinguished three types of environment (3): home, restaurant/pub or outdoors. Number of birds (4) was recorded as one or more bird individuals engaged in alcohol consumption. Birds (5) were divided into three categories: wild, pet and poultry species. Types of channel (6): personal, news or humorous. Birds were recorded to species level (7) wherever possible; however, in some cases this was not possible. Social response (8) was measured as the numbers of thumbs up/down ratings but this was possible only for YouTube material. Some variables contained missing data, and hence sample size may differ between analyses. 

As an approximation of the popularity of particular bird species, we used the main platform of Google for species-specific searches. We scored species according to the number of results provided by Google browser where the searched phrase appeared (the value at the top of the first search page). We used English names of birds and conducted a search for each species independently, and assumed that the Google score relates to public appeal and popularity of each species [20]. A binary logistic regression model of social response (thumbs up/down) to YouTube videos was carried out on six potential explanatory variables ((1)–(6)) described above. Because of gaps in some variables this model was based on 146 videos with complete data for all variables. Spearman rank correlation was used to compare Google species-specific scores with YouTube hits and with numbers of YouTube videos. All analyses were undertaken in Minitab18.

### Visualization of Alcohol Consumption on Avian Phylogenetic Tree

**Figure 1:**
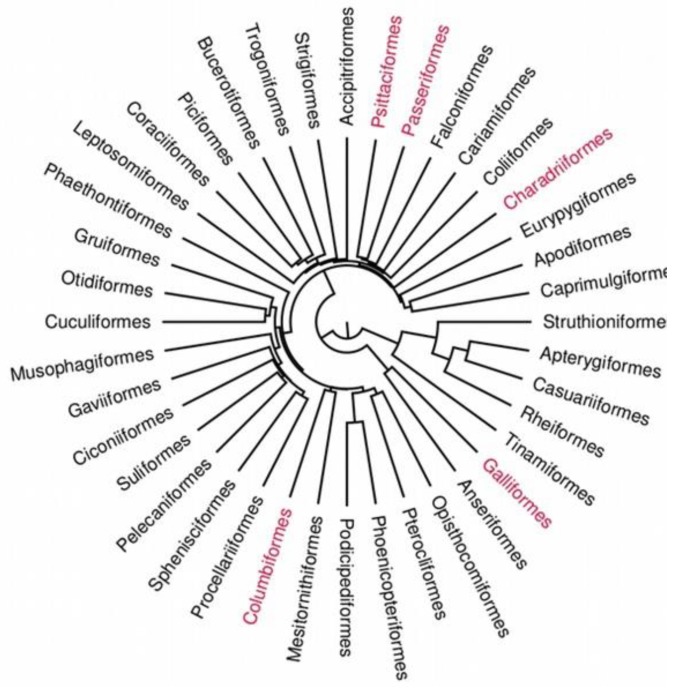
Visualization of alcohol consumption on an avian phylogenetic tree. Orders identified drinking alcohol are marked in red.

To check how alcohol consumption behaviour is distributed over the avian phylogenetic tree we reconstructed an evolutionary history of extant avian orders (Figure 1) using the online tool available at http://birdtree.org/ [21]. The maximum credibility tree was built from 100 randomly generated trees based on a Hackett backbone [22] (we chose one species from each order to do this). We determined the maximum clade credibility tree using the TreeAnnotator tool v. 1.8.2 in the BEAST software package v. 1.8.2 [23].

## 3. Results

### 3.1. Taxonomic Representation

A total of at least 55 species were identified, 11 in the scientific literature (Table 1) and 47 in videos (Table 2). The two data sources differed in the reported species. Only three species occurred in both data sources: cedar waxwing *Bombycilla cedrorum*, Bohemian waxwing *Bombycilla garrulus*, and European starling *Sturnus vulgaris*. 

Drinking alcohol was noted among five independent clades and avian orders distributed across the phylogenetic tree suggesting that the suggested origin of this convergent behaviour is mainly human influence (Figure 1).

**Figure 2:**
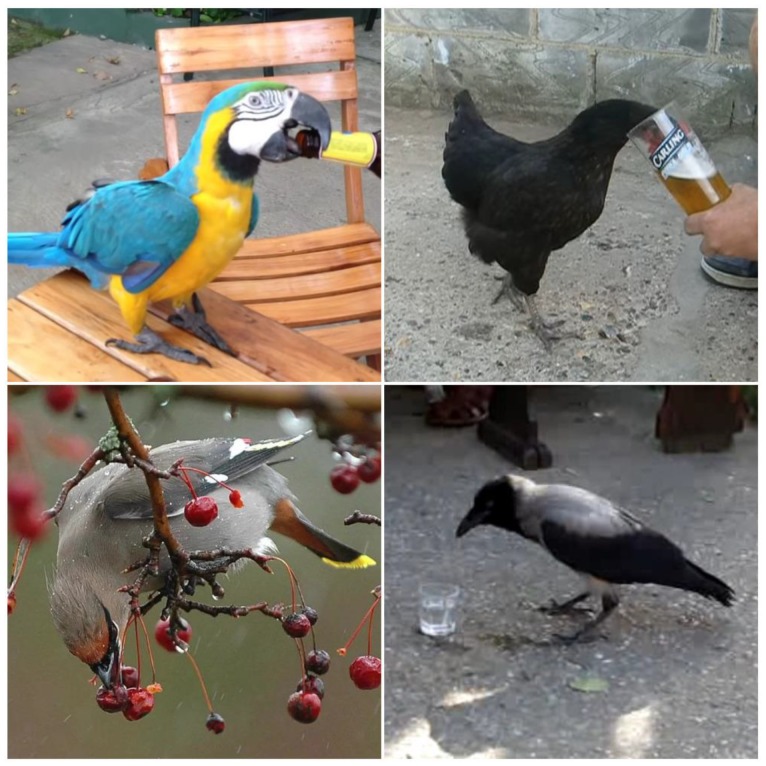
Examples from YouTube of birds drinking alcoholic beverages: (**a**) blue-and-yellow macaw *Ara ararauna* drinking beer in a pub in Columbia, https://youtu.be/nXZSVfT7UwI; (**b**) hen *Gallus gallus* drinking beer, https://youtu.be/ubqHeIiyVf4; (**c**) Bohemian waxwing *Bombycilla garrulus* eating ripe berries and subsequently having problems with flight, Canada, https://youtu.be/d_NqGjV3l_8; (**d**) hooded crow *Corvus cornix* drinking vodka, Hungary, https://youtu.be/xlq6GCekPNA.

For YouTube videos it was possible to do more detailed analyses, and among 51 taxonomic categories (47 species and in 4 cases birds diagnosed to higher taxa level), 22 categories (43.1%) were wild birds, 27 were pets (52.9%), and 2 (3.9%) were poultry. Interestingly, data included 29 parrots and 6 corvids, and the highest number of videos was of the very common pet, the budgerigar *Melopsittacus undulatus*. Among wild birds, the most popular was the hooded crow *Corvus cornix* (Figure 2). 

For 47 identified species, we found a highly significant positive Spearman correlation between the Google score and YouTube hits (r_s_ = 0.739, *p* < 0.001), and a significant positive Spearman correlation between Google score and number of YouTube videos (r_s_ = 0.344, *p* = 0.018). 

Where recorded, 70.4% of the YouTube videos involved beer. Our interpretation of the videos was that alcohol was provided unintentionally in 58.4% of cases. The environment was recorded as home for 53.4% of videos, restaurant/pub for 7.4% and outdoors for 39.2%. Single birds accounted for 89.4% of videos. The type of bird was recorded as wild for 22% of videos, pet for 67.2% and poultry for 10.7%. The type of channel was personal for 88.3% of videos, news for 5.0% and humorous for 6.7%.

### 3.2. Factors Affecting Human Perception of Drinking Birds

Based on YouTube videos it was possible to check human perception of birds drinking through their preferred emotions (thumbs up or thumbs down) to the videos (Table 3). People preferred videos when the bird drank beer rather than stronger alcohol (*p* = 0.001), when the drink was provided unintentionally (*p* < 0.001), when videos were recorded at home rather than in restaurants/pubs or outdoors (*p* < 0.001), when only a single bird was involved (*p* < 0.001), when videos were of wild birds rather than of pets or poultry (*p* < 0.001) and when the video came from a news channel (*p* < 0.001). 

## 4. Discussion

Many different bird species were recorded consuming alcoholic drinks; in the majority of cases intentionally or unintentionally (for example when left on a table) provided by humans. This is a different situation from when they occasionally consume ethanol in the wild, mainly from fermented fruit, especially berries [7,8]. Interestingly, only three species were recorded consuming alcohol in both the scientific literature and video data sources, and all three (cedar and Bohemian waxwings, and European starling) are classical subjects for the study of the effects of alcohol on birds. These studies combined both experimental and observational data [8,10,24] and even necropsy examination after collision of drunken birds with solid objects, such as picture windows, plexiglass or fences [25]. Enlarging the list of bird species that consume alcohol was possible due to the use of both classical scientific data and also the more numerous information available from YouTube and other video sources. Two groups of species, not previously reported in the scientific literature, often occurred in YouTube videos, namely parrots and corvids. Both of these groups of birds are very intelligent, with a broad spectrum of interactions with humans, including supplementary feeding, mobbing, food stealing and accessibility to novel food [19,26,27]. They include charismatic, even celebrity, species and individuals, such as a grey parrot named Alex [17,18], and in consequence they may simply be recorded more often. Moreover, the distribution of orders across the avian phylogenetic tree suggests that drinking alcohol was strongly affected by human influence, rather than by phylogenetic relationships.

Probably a difference in charisma between species [18], as well as differences in geographical distribution of species used in previous scientific studies, are the cause of the absence of other subjects of scientific research in YouTube videos [28]. Recent access to the internet has extended temporal and spatial boundaries and made the world a global village where electronic, especially social, media has rapidly increased the accessibility and immediacy of information [29], but with some restrictions. However, once again, data collected from social media, including YouTube, if used with caution, can be useful for understanding the behaviour of animals [15,16].

A review of birds using ethanol provokes the question: why do they do it? Ethanol contains almost twice the mass-specific energy of glucose, and this explanation was used to understand the choice of ripe fruit by birds [6,7,28]. However, alcohol in the form of drinks is not normally used by birds, although it can in principle be similar to tree sap [8]. We do consider that alcohol may produce problems for individuals, such as increasing the probability of collisions with buildings or generally a slower reaction to predator presence, although this is only speculation in the light of the behaviour of some mammals [1,2,3]. Human-provided drinks are probably not a very important source of energy for birds, because they are consumed by wild birds only very occasionally. The consumption of alcohol is probably encouraged by modified/broken barriers with humans which even give birds access to novel, mainly junk, food [30]. Moreover, the high representation of drinking parrots is not only because they are common pets and thus recorded on amateur movies, but also because they have a very strong metabolism response to potential poisons, including alcohols and phenols [31]. Generally, ethanol consumption is detrimental to the fitness of organisms since it increases susceptibility to predation [32,33] by interfering with motor skills resulting in a significant loss of coordination [34]. But perhaps drinking birds are then easier to record, and people appear to enjoy the spectacle. 

This is also confirmed by more positive than negative reactions of video watchers; of the 6353 opinions expressed on the YouTube videos summarized here, 84.1% were positive (thumbs up). Obviously, people’s reactions to drinking birds were modified by some factors, namely, type of alcoholic beverage, interaction with humans, environment, number of birds in the video, type of bird and type of channel. Moreover, emotions given by YouTube users are not clear classifications, and some are given very quickly by the observer without much thought [35]. Videos showing consumption of beer are probably liked more because beer is often linked to nature and fun activities in advertisements [36]. Beer consumers may have a different view of fun, nature and also social skills [37]. A more positive reaction to videos on wild birds is probably related to the special relationship between people and wildlife [17,18], although interactions with pets are sometimes also spectacular [38]. A little surprising is the lower number of positive thumbs up for poultry, but maybe because some of these involve forced consumption of alcohol. It is interesting that news channel videos on YouTube got a more positive reaction than social media. This may be related to the quality of the videos, general publicity (links from newspapers and blogs), but especially with one particular case, in which a drunk kererū pigeon, *Hemiphaga novaeseelandiae*, was even voted as New Zealand bird of the year 2018, and it generally is a species with a strong biocultural presence (SI) [39].

It was not possible to distinguish how different kinds of alcoholic drinks affected birds, and which they chose to drink. Do they simply consider them a source of water? It is possible that they are attracted partially by the colour of the beverage, as was described for chicken [40], a poultry species also represented in our sample. Fruits in the wild have different colours according to ethanol level and have different bird preferences [6,30]. However, due to the sample size limitation of the data, this interesting hypothesis is impossible to test directly. Many questions can be answered in rigorous experimental tests, similar to those already carried out on ethanol and birds (Table 2) [5,10,11], but we strongly recommend also including parrots, pets very often represented in the social media sample. These are generally easy to rear in cages and the results (both positive, as well as negative, are important) could be very interesting, not only to researchers, but also to the general public.

## 5. Conclusions

We documented that birds drinking alcohol, especially from alcoholic beverages provided by humans, is much more common than currently documented in the scientific literature. Data from YouTube videos suggest that drinking alcohol is probably a widely distributed phenomenon supported by human behaviour. Parrots feature often among drinking birds, and we think that this group of species should be subject to more advanced study under laboratory conditions. 

## Figures and Tables

**Table 1:** Interactions between birds and alcohol provided in different forms reported in the scientific literature. Sources are arranged from oldest to newest.

Common Name	Scientific Name	Type of Data	Alcohol Provided by	Source
Bohemian waxwing	*Bombycilla garrulus*	Experiment	berries	7
Eurasian bullfinch	*Pyrrhula pyrrhula*	Experiment	berries	7
European starling	*Sturnus vulgaris*	Experiment	berries	7
European greenfinch	*Chloris chloris*	Experiment	berries	7
European starling	*Sturnus vulgaris*	Experiment	ingested	9
Bohemian waxwing	*Bombycilla garrulus*	Observation	berries	20
Pine grosbeak	*Pinicola enucleator*	Observation	berries	20
Yellow-vented bulbul	*Pycnonotus xanthopygos*	Experiment	ripe fruit	5
Cedar waxwing	*Bombycilla cedrorum*	Necropsy after collision	berries	21
Zebra finch	*Taeniopygia guttata*	Experiment	cloacal injection	10
Woodpeckers	*Picidae*	Suggestion only	tree sap	8
Cape white-eye	*Zosterops virens*	Experiment	overripe fruits	6
Speckled mousebird	*Colius striatus*	Experiment	overripe fruits	6
Red-winged starling	*Onychognathus morio*	Experiment	overripe fruits	6

**Table 2:** List of bird species recorded in YouTube videos consuming alcoholic beverages. Birds are ordered according to the number of YouTube videos. Birds already mentioned in the scientific literature (Table 1) in the context of alcohol consumption are shaded grey. The type of bird (wild, pet or poultry) and the Google score (number of results (hits) given at the top of the first search page) are also presented.

Common Name	Scientific Name	Type	YouTube (# Videos)	Google (# Hits)
Budgerigar	*Melopsittacus undulatus*	pet	50	2.37 M
Chicken	*Gallus gallus*	poultry	17	1500M
Cockatiel	*Nymphicus hollandicus*	pet	11	20.1 M
Aratinga	*Aratinga* spp.	pet	7	1.53 M
Grey parrot	*Psittacus erithacus*	pet	7	55 M
Orange-winged Amazon	*Amazona amazonica*	pet	6	9.83 M
Blue-and-yellow macaw	*Ara ararauna*	pet	6	33.7 M
Green-cheeked parakeet	*Pyrrhura molinae*	pet	6	27.8 M
Hooded crow	*Corvus cornix*	wild	5	5.12 M
American robin	*Turdus migratorius*	wild	4	529 M
Turquoise-fronted Amazon	*Amazona aestiva*	pet	3	325 k
Pigeons	*Columbidae*	wild	3	92.5 M
Common raven	*Corvus corax*	wild	3	63.7M
Rose-ringed parakeet	*Psittacula krameri*	pet	3	21.8M
Rainbow lorikeet	*Trichoglossus moluccanus*	pet	3	1.39M
Rosy-faced lovebird	*Agapornis roseicollis*	pet	2	489k
Mallard	*Anas platyrhynchos*	wild	2	46.9M
Red-and-green macaw	*Ara chloropterus*	pet	2	53.9M
Cedar waxwing	*Bombycilla cedrorum*	wild	2	3.08M
White cockatoo	*Cacatua alba*	pet	2	593k
Rock dove	*Columba livia*	wild	2	236M
Green-thighed parrot	*Pionites leucogaster*	pet	2	15.7M
Coconut lorikeet	*Trichoglossus haematodus*	wild	2	206k
Sparrows	*Passer* spp.	wild	1	133M
Red-legged partridge	*Alectoris rufa*	wild	1	1.01M
Yellow-crowned Amazon	*Amazona ochrocephala*	pet	1	5.31M
Yellow-headed Amazon	*Amazona oratrix*	pet	1	48.8M
Amazon	*Amazona* spp.	pet	1	63.6M
Hyacinth macaw	*Anodorhynchus hyacinthinus*	pet	1	1.41M
Bohemian waxwing	*Bombycilla garrulus*	wild	1	532k
Sulphur-crested cockatoo	*Cacatua galerita*	pet	1	4.27M
Salmon-crested cockatoo	*Cacatua moluccensis*	pet	1	263k
Little corella	*Cacatua sanguinea*	pet	1	11.6M
Yellow-crested cockatoo	*Cacatua sulpharea*	pet	1	5.35M
Common wood pigeon	*Columba palumbus*	wild	1	25.3M
Carrion crow	*Corvus corone*	wild	1	2.83M
Rook	*Corvus frugilegus*	wild	1	18.2M
Western jackdaw	*Corvus monedula*	wild	1	869k
Galah	*Eolophus roseicapilla*	pet	1	4.71M
Chopi blackbird	*Gnorimopsar chopi*	wild	1	135M
Venezuelan troupial	*Icterus icterus*	wild	1	30.2k
European herring gull	*Larus argentatus*	wild	1	9.84M
Turkey	*Meleagris gallopavo*	poultry	1	342M
Monk parakeet	*Myiopsitta monachus*	pet	1	605k
Nestor kea	*Nestor notabilis*	wild	1	330k
Grey jay	*Perisoreus canadensis*	wild	1	381M
Western tanager	*Piranga ludoviciana*	wild	1	2.63M
Red-masked parakeet	*Psittacara erythrogenys*	pet	1	351k
Alexandrine parakeet	*Psittacula eupatria*	pet	1	252k
European starling	*Sturnus vulgaris*	wild	1	9.81M
Red-collared lorikeet	*Trichoglossus rubritorquis*	pet	1	483k

**Table:** **Deviance Table**.

Source	DF	Adj. Dev.	Adj. Mean	Chi-Square	*p*-Value
Regression	9	698.17	77.575	698.17	<0.001
Alcohol Type	1	10.82	10.816	10.82	0.001
Interaction	1	54.92	54.920	54.92	<0.001
Environment	2	39.92	19.958	39.92	<0.001
Number	1	18.15	18.146	18.15	<0.001
Bird type	2	282.15	141.076	282.15	<0.001
Channel	2	27.65	13.825	27.65	<0.001
Error	136	591.58	4.350		
Total	145	1289.75			

**Table:** **Coefficients**.

Term	Coef	SE
Constant	3.738	0.181
**Alcohol Type**		
Beer	0.000	0.000
Other	−0.356	0.107
**Interaction**		
Unintentional	0.000	0.000
Intentional	−0.824	0.112
**Environment**		
House	0.000	0.000
Restaurant/pub	−0.984	0.188
Outdoors	−0.914	0.163
**Number**		
One	0.000	0.000
More than one	−0.801	0.188
**Bird**		
Wild	0.000	0.000
Pet	−1.348	0.140
Poultry	−1.622	0.103
**Channel**		
Personal	0.000	0.000
News	1.077	0.214
Humorous	0.0980	0.0917

## Supplementary Materials

The following are available online, Examples of mass-media articles on drinking birds: https://www.dailyedge.ie/mass-bird-deaths-caused-by-alcohol-68081-Jan2011/; https://bringmethenews.com/minnesota-lifestyle/drunk-birds-are-causing-problems-in-a-northern-minnesota-city; https://www.fakt.pl/wydarzenia/swiat/pijane-mewy-na-plazach-w-anglii-opily-sie-piwem/p9pvm62; https://www.telegraph.co.uk/news/2018/07/05/drunk-seagulls-taken-rspca-centres-drinking-discarded-alcohol/; https://www.australiangeographic.com.au/topics/wildlife/2011/10/drunk-birds-inebriation-in-the-wild/; https://www.theguardian.com/world/2018/oct/15/new-zealand-bird-of-the-year-drunk-gluttonous-kereru-pigeon-wins.

## References

[1] Dudley R. (2014). The Drunken Monkey: Why We Drink and Abuse Alcohol.

[2] Levey D.J. (2004). The evolutionary ecology of ethanol production and alcoholism. Integr. Comp. Biol..

[3] Krzyżewski J. (2012). Alkohol w świecie zwierząt. Kosmos.

[4] Scanes C.G., Pierzchala-Koziec K. (2018). Perspectives on Endogenous Opioids in Birds. Front. Physiol..

[5] Mazeh S., Korine C., Pinshow B., Dudley R. (2008). The influence of ethanol on feeding in the frugivorous yellow-vented bulbul (Pycnonotus xanthopygos). Behav. Proces..

[6] Zungu M.M., Downs C.T. (2017). Effects of ethanol on fruit selection by frugivorous birds. Afr. Zool..

[7] Eriksson K., Nummi H. (1982). Alcohol accumulation from ingested berries and alcohol metabolism in passerine birds. Ornis Fenn..

[8] Rasal V. (2015). Sap-Drinking by Birds on Tapped Indian Date Palm Phoenix sylvestris. J. Bombay Nat. Hist. Soc..

[9] Suhonen J., Jokimäki J. (2015). Fruit removal from rowanberry (Sorbus aucuparia) trees at urban and rural areas in Finland: A multi-scale study. Land. Urban Plan..

[10] Prinzinger R., Hakimi G.A. (1996). Alcohol resorption and alcohol degradation in the European Starling Sturnus vulgaris. J. Orn..

[11] Olson C.R., Owen D.C., Ryabinin A.E., Mello C.V. (2014). Drinking songs: Alcohol effects on learned song of zebra finches. PLoS ONE.

[12] Knutie S.A., Chaves J.A., Gotanda K.M. (2019). Human activity can influence the gut microbiota of Darwin’s finches in the Galapagos Islands. Mol. Ecol..

[13] Dennis J.V. (1987). If you drink, don’t fly: Fermented fruit and sap can inebriate birds. Birder’s World.

[14] Tryjanowski P. (2018). Wine and Birds.

[15] Dylewski Ł., Mikula P., Tryjanowski P., Morelli F., Yosef R. (2017). Social media and scientific research are complementary—YouTube and shrikes as a case study. Sci. Nat..

[16] Measey J., Basson A., Rebelo A.D., Vimercati G., Louw M., Mohanty N.P. (2019). Why have a pet amphibian? Insights from YouTube. Front. Ecol. Evol..

[17] Robinson S.K. (2019). Bird niches in human culture and why they matter. Proc. Natl. Acad. Sci. USA.

[18] Schuetz J.G., Johnston A. (2019). Characterizing the cultural niches of North American birds. Proc. Natl. Acad. Sci. USA.

[19] Lefebvre L., Reader S.M., Sol D. (2004). Brains, innovations and evolution in birds and primates. Brain Behav. Evol..

[20] Żmihorski M., Dziarska-Pałac J., Sparks T.H., Tryjanowski P. (2013). Ecological correlates of the popularity of birds and butterflies in Internet information resources. Oikos.

[21] Jetz W., Thomas G.H., Joy J.B., Hartmann K., Mooers A.O. (2012). The global diversity of birds in space and time. Nature.

[22] Hackett S.J., Kimball R.T., Reddy S., Bowie R.C., Braun E.L., Braun M.J., Chojnowski J.L., Cox W.A., Han K.L., Harshman J. (2008). Phylogenomic study of birds reveals their evolutionary history. Science.

[23] Drummond A.J., Rambaut A. (2007). BEAST: Bayesian evolutionary analysis by sampling trees. BMC Evol. Biol..

[24] Stephen L.J., Walley W.J. (2000). Alcohol intoxication contributing to mortality in Bohemian waxwings and a pine grosbeak. Blue Jay.

[25] Kinde H., Foate E., Beeler E., Uzal F., Moore J., Poppenga R. (2012). Strong circumstantial evidence for ethanol toxicosis in Cedar Waxwings (Bombycilla cedrorum). J. Orn..

[26] Emery N.J., Clayton N.S. (2005). Evolution of the avian brain and intelligence. Curr. Biol..

[27] Sol D., Duncan R.P., Blackburn T.M., Cassey P., Lefebvre L. (2005). Big brains, enhanced cognition, and response of birds to novel environments. Proc. Natl. Acad. Sci. USA.

[28] Thelwall M. (2018). Social media analytics for YouTube comments: Potential and limitations. Int. J. Social Res. Methodol..

[29] Valcanis T. (2011). An iPhone in every hand: Media ecology, communication structures, and the global village. ETC Rev. Gen. Semant..

[30] Willson M.F., Whelan C.J. (1990). The evolution of fruit color in fleshy-fruited plants. Am. Nat..

[31] Coogan S.C., Raubenheimer D., Zantis S.P., Machovsky-Capuska G.E. (2018). Multidimensional nutritional ecology and urban birds. Ecosphere.

[32] Masello J.F., Martínez J., Calderón L., Wink M., Quillfeldt P., Sanz V., Theuerkauf J., Ortiz-Catedral L., Berkunsky I., Brunton D. (2018). Can the intake of antiparasitic secondary metabolites explain the low prevalence of hemoparasites among wild Psittaciformes?. Parasit. Vect..

[33] Janzen D.H. (1977). Why fruits rot, seeds mold, and meat spoils. Am. Nat..

[34] Sánchez F., Melcón M., Korine C., Pinshow B. (2010). Ethanol ingestion affects flight performance and echolocation in Egyptian fruit bats. Behav. Proces..

[35] Chen Y.L., Chang C.L., Yeh C.S. (2017). Emotion classification of YouTube videos. Dec. Supp. Syst..

[36] Pettigrew S., Roberts M., Pescud M., Chapman K., Quester P., Miller C. (2012). The extent and nature of alcohol advertising on Australian television. Drug Alco. Rev..

[37] Klatsky A.L., Armstrong M.A., Kipp H. (1990). Correlates of alcoholic beverage preference: Traits of persons who choose wine, liquor or beer. Br. J. Add..

[38] Wilkins A.M., McCrae L.S., McBride E.A. (2015). Factors affecting the human attribution of emotions toward animals. Anthrozoös.

[39] Lyver P.O., Ruru J., Scott N., Tylianakis J.M., Arnold J., Malinen S.K., Bataille C.Y., Herse M.R., Jones C.J., Gormley A.M. (2018). Building biocultural approaches into Aotearoa–New Zealand’s conservation future. J. Roy. Soc. N. Zeal..

[40] Marples N.M., Roper T.J. (1996). Effects of novel colour and smell on the response of naive chicks towards food and water. Anim. Behav..

